# Breast cancer risk associated with different HRT formulations: a register-based case-control study

**DOI:** 10.1186/1472-6874-6-13

**Published:** 2006-09-12

**Authors:** Juergen C Dinger, Lothar AJ Heinemann, Sabine Möhner, Do Minh Thai, Anita Assmann

**Affiliations:** 1 Centre for Epidemiology & Health Research Berlin, Invalidenstr. 115, 10115 Berlin, Germany

## Abstract

**Background:**

Previous epidemiological studies have inconsistently shown a modestly increased breast cancer risk associated with hormone replacement therapy (HRT). Limited information is available about different formulations – particularly concerning different progestins.

**Methods:**

A case-control study was performed within Germany in collaboration with regional cancer registries and tumor centers. Up to 5 controls were matched breast cancer cases. Conditional logistic regression analysis was applied to estimate crude and adjusted odds ratios (OR) and 95% confidence intervals (95% CI). Stratified analyses were performed to compare the risk of different estrogens, progestins, and combinations.

**Results:**

A total of 3593 cases of breast cancer were identified and compared with 9098 controls. The adjusted overall risk estimate for breast cancer (BC) associated with current or past use of HRT was 1.2 (1.1–1.3), and almost identical for lag times from 6 months to 6 years prior to diagnosis. No significant trend of increasing BC risk was found with increasing duration of HRT use, or time since first or last use in aggregate. Many established BC risk factors significantly modified the effect of HRT on BC risk, particularly first-degree family history of BC, higher age, lower education, higher body mass index (BMI), and never having used oral contraceptives (OCs) during lifetime.

Whereas the overall risk estimates were stable, the numbers in many of the sub-analyses of HRT formulation groups (estrogens, progestins, and combinations) were too small for strong conclusions. Nevertheless, the BC risk seems not to vary much across HRT formulation subgroups. In particular, no substantial difference in BC risk was observed between HRT containing conjugated equine estrogens (CEE) or medroxyprogesterone acetate (MPA) and other formulations more common in Europe.

**Conclusion:**

The BC risk of HRT use is rather small. Low risk estimates for BC and a high potential for residual confounding and bias in this observational study do not permit causal conclusions. Apparently, there is not much variation of the BC risk across HRT formulations (estrogens, progestins). However, the small numbers and the overlapping nature of some of the subgroups suggest cautious interpretation.

## Background

Discussion surrounding the role of steroid hormone formulations in carcinogenesis is complex, i.e. providing arguments for both benefits and risks in short and long-term use [1] In 1968 discussion of benefits and adverse effects was launched in large part following publication of the study by the Royal College of General Practitioners [2]. Specific discussion of adverse effects of estrogens or estrogen/progestin combinations used for the alleviation of vasomotor symptoms of peri- and post-menopausal women – called Hormone Replacement Therapy (HRT) – hit its peak with the publication of the Women's Health Initiative (WHI) Study [3] and the Million Women Study (MWS) [4].

In addition to their efficacy for the treatment of vasomotor symptoms benefits of HRT have been shown by observational studies and clinical trials for the prevention of osteoporosis and fractures [5,6], as well as lower risk of endometrial and colorectal cancer [4,5,7]. Furthermore, many observational studies of HRT have been associated with a 40–50% lower risk of acute cardiovascular events in women using hormone replacement therapy (less often for combinations with progestins) [8,9]. However, this was not confirmed by the WHI study. It is not clear whether HRT has an impact on real primary prevention of cardiovascular diseases [10,11].

The overall risk of breast cancer associated with HRT has shown variation, but has mainly been reported as significantly increased but well under a risk estimate of 2.0 [12,13]. In previous publications, we have shown no increased risk for gynecologic cancers at least – and in fact even weak evidence for preventive properties of sex steroids in young women using oral contraceptives [14-16]. The WHI study results [3] published in 2002 for breast cancer (BC) and HRT use that showed a significantly increased association with estrogen plus progestin treatment based on combined hormonal therapy of conjugated equine estrogens (CEE) with medroxyprogesterone acetate (MPA). This combination is the most commonly used HRT in North America. In Europe, however, the most common HRT preparations are based on estradiol.

The objective of this publication is to present data on BC risk associated with HRT use in Germany were CEE/MPA is less frequently used than formulations based on estradiol and a large series of progestin other than MPA. This paper is also focused on the question of different BC risk in different HRT formulation sub-groups.

## Methods

### Objectives

The objective of this case-control study was to provide evidence regarding the presence or absence of differential effects of HRT formulations used in Europe on cancer risks or benefits in collaboration with German cancer registries and tumor centers. The study was planned in early 2003 (performed 2004–2005), and was justified by the working hypothesis that the results obtained with the HRT formulation CEE/MPA might be not generalizable for Europe with HRTs of other estrogens and progestins. This study on HRT was made possible by earlier collaborative research with German cancer registries and tumor centers in the field of gynecological cancer and exogenous steroid hormone use [14-16]. The study was designed as a case-control study with lifetime history of exposure and information about patient's characteristics as well as risk factors for BC.

Cancer registries in Schleswig Holstein, Hamburg, and the common registry for the "five new states" (former East Germany) participated in this study, as well as clinical tumor centers throughout Germany.

The exposure data (sex steroid hormone use) were recorded by month and year of exposure (type, brand name) as well as the date of diagnosis of cancer. The information was compiled primarily based on a questionnaire. The cancer registries distributed the questionnaires to the breast cancer cases. The participation was voluntary and a consent form was required.

The study protocol and questionnaire were approved by the relevant Ethical Committee and the Office of Data Privacy.

The Centre for Epidemiology & Health Research Berlin (ZEG Berlin) coordinated the study. A number of university-based and other collaborators contributed with ideas/proposals to the study, specific sub-studies, evaluation, and interpretation.

### Cases and controls

Eligible cases were those of histologically confirmed breast cancer (ICD 10: C50) diagnosed until 2004 inclusively in women of all age groups. The vast majority got their breast cancer diagnosed between 2000 and 2004, but 7% before 2000. Subjects had to be alive and in sufficiently good shape to complete the questionnaire in order to qualify as cases for the study. Cases were considered non-eligible if they showed a history of other malignant tumors or missing time variables (diagnosis, exposure).

An initial total of 3717 BC cases were identified. Of these, 124 cases were excluded for different reasons: in 66 cases the diagnosis was not fully confirmed, and 58 had a uterine or ovarian cancer before or in the same time window as BC. Thus 3593 BC cases were used for this analysis.

The participants of the German Cohort Study on Women's Health, that were shown to be representative for German Women in many aspects [14-17], were used as pool for controls (if without history of malign tumors). Up to five controls were matched for each BC case for same age as case (year of birth +/- two years) and residency (Bundesland) as matching criteria. Of a total of 18,898 potential controls, we found 9098 matched controls for 3593 breast cancer cases.

### Data collection, variables and database preparation

Time-related information on lifetime history of hormone use as well as data on reproductive life, lifestyle pattern, conditions/diseases, and some other factors were obtained via a self-administered postal baseline questionnaire. Other studies have shown that the recall of hormone use history (type and duration of use) can be reliably obtained from women even for periods far in the past [18-20]. The same data were obtained from cases and controls. Many consistency checks were made. Telephone inquiries with respondents were conducted to improve the data quality, if questionnaire data were inconsistent, not sufficiently clear, or missing.

The type of HRT formulation was classified into five broad categories: estrogen/progestin alone, combination type, estrogen type, progestin type, and CEE/MPA category. The lifetime history of HRT use was divided into multiple sub-groups: "only HRTs" (estrogen only and progestagen only), combination form (sequential, continuous-combined, and all E+P together), according to the estrogen type (estradiol, E2, EE, CEE), according to progestins (NETA, norgestrel, LNG, MPA, CMA, CPA, medrogestone, dydrogesterone, hydroxyprogesterone desogestrel, lynestrenol, progesterone, dienogest, tibolone, raloxifene), and mutually exclusive categories of CEE/MPA combinations (CEE+MPA, CEE or MPA, no CEE & no MPA). Each participant who had ever used HRT was classified in accordance with these categories, which are *not* mutually exclusive but rather overlapping, i.e. women might have used more than one of the HRT formulations during lifetime and therefore counted more than once. In many of these subgroups, we expected to run out of numbers because of low exposure prevalence.

### Index dates

We considered an important possible bias for analysis using the logistic regression model: The possibility cannot be excluded that prior to the final diagnosis of breast cancer, the women and/or their physicians might have been aware of "warning signs" (e.g. breast lumps, suspicious areas in mammograms) that may have led to a decision not to use HRT. In addition, knowledge about HRT might have led to more intense screening/diagnostics for early forms of cancer. We therefore introduced several index dates (= different lag times): For example, we disregarded HRT use and other exposure information in the last six months prior to the final cancer diagnosis across all analyses, i.e. for both cases and controls. A series of further index dates was introduced, such as 2, 4, and 6 years, i.e. all exposure-related information prior to these dates was ignored in the respective analyses. It may be debated whether 6 years prior to diagnosis is too long a period to disregard information, because HRT use during the 6 years before diagnosis might well have promoted tumor growth leading to earlier diagnosis. Attempts to establish whether this is a problem were undertaken by analyzing the effect of increasing length of time since first and last HRT use as well as duration of use.

### The analytic model

The main analysis was done with conditional logistic regression. Crude and adjusted odds ratios (OR) were reported with 95% confidence intervals (95% CI). Adjustment was done for BMI, family history of breast/uterine/ovarian cancer, child-bearing history, age at first live birth, duration of breast-feeding, age at menarche, OC use, and education. If values were missing for the adjustment variables, we used automatically imputed figures such as modal values. For sub-group analyses where the matching effect got lost due to other group definitions (HRT formulation subgroups), we adjusted for age and residency to replace the matching. We were not able to use age at menopause as co-variable because it could not be reliably determined for a high proportion of participants.

The number of cancer cases was – despite the large total number – too small for many sub-analyses (e.g. HRT formulation subgroups), we refrained from complex adjustment to prevent unstable risk estimates, and i.e. we adjusted only for age and residency. Moreover, we did not calculate risk estimates if any cell of the "two-by-two" table contained less than 5 women.

We analyzed the impact of duration of HRT use compared to never-use (categories: never, <2 years, 2–4 years, 5–7 years, and 8+ years). The time elapsed from first HRT use to the manifestation of BC was categorized as follows: never-use, 1–4 years, 5–9 years, 10–14 years, 15+ years. The respective categories for time since last use were: never-use, <1 year, 1–2 years, 3–4 years, and 5+ years ago. Short use (less than 1 year) might be differently recalled by women with cancer cases versus those without cancer (if longer ago).

It was not possible to define mutually exclusive exposure categories to analyze individual HRT formulations. Use of HRT formulations partly overlaps across life histories due to switching pattern. Furthermore, it was not feasible to define sufficiently large groups with exclusive use of only one specific hormonal formulation group. Therefore, comparison of risk estimates across exposure groups provides only an impression as to whether there are substantial differences in cancer risk.

We abstained also from defining categories of HRTs or compounds "longest used during lifetime", because of the arbitrary character of the decision as well as the assumed incompatibility for what "longest" might mean across different compounds.

The statistical packages SAS 9.1 and STATA 8.0 were used.

## Results

### Case/non-case characteristics

Table 1 describes the breast cancer cases and the matched controls. The mean age of the cancer cases and matched controls was similar as was age at menarche and age at first live birth. The educational level was slightly higher in the control group than case group.

**Table 1:** Characteristics of breast cancer cases and controls.

	Breast cancer N = 3593		BC controls N = 9098	
Age at diagnosis (mean, SD)	54.6	9.3	50.6	10.1
	N	%	N	%
Education				
University level	1066	29.7	3215	35.3
Lower education	2483	69.1	5817	63.9
Age at menarche				
< 13 years	1926	53.6	4870	53.5
13 + years	1583	44.1	3998	43.9
Child bearing history				
0–1 child	1524	42.4	3358	36.9
2+ children	1992	55.4	5648	62.1
Age at first live birth				
<= 22 years	1502	41.8	3821	42.0
> 22 years	1626	45.3	4276	47.0
Nulliparous & no live birth	367	10.2	840	9.2
Breast-feeding				
never	1924	53.6	3957	43.5
1 month and more	1592	44.3	5047	55.5
OC use				
never	1079	30.0	1805	19.8
ever	2508	69.8	7271	79.9
HRT use				
never	2080	57.9	5910	65,0
ever	1513	42.1	3188	35.0
Family history of breast cancer				
No	2453	68.3	8400	92.3
Yes	550	15.3	649	7.1
Body mass index				
<25	1415	39.4	4470	49.1
25 +	2152	59.9	4508	49.6

Other descriptive data in Table 1 refer to known risk factors for breast cancers that were used as adjustment factors in the analyses. Controls had more children, more often breast-feeding, had more frequently a history of OC use, but less frequently ever used HRT than cases. The BMI was somewhat lower in controls and family history of breast cancer was less prevalent than in cases. Most of these variables were used to control for confounding in the main analyses of BC risk.

### Ever-never HRT use by index date

We analyzed the cancer risk of HRT ever use vs. never use (Table 2) with conditional logistic regression with different lag-time (index dates) between HRT-exposure and cancer diagnosis. A marginally increased risk (OR 1.2) was observed for breast cancer with little variation across all index dates, i.e. allowing a lag-time from 6 months to 6 years between HRT exposure and breast cancer diagnosis. Little difference was also found between crude and adjusted relative risk estimates. The adjusted risk estimates obtained in the matched and unmatched did not differ (data not shown).

**Table 2:** Risk of breast cancer associated with HRT: Ever- use of HRT use vs. never use analyzed with conditional logistic regression by increasing lag-time (index dates).

**Breast cancer**		**Conditional logistic regressions by index dates: OR (95% CI)**				
		**No index date**	**0.5**	**2.0**	**4.0**	**6.0**
	**Crude**	1.1 (0.99–1.2)	1.1 (0.99–1.2)	1.1 (0.99–1.2)	1.2 (1.0–1.3)	1.2 (1.0–1.3)
	**Adjusted**	1.2 (1.1–1.3)	1.2 (1.0–1.3)	1.2 (1.0–1.3)	1.2 (1.1–1.4)	1.2 (1.1–1.4)

Conditional logistic regression analysis [Odds ratios (OR) and 95% confidence intervals]; adjustment for BMI, family history of breast cancer, childbearing history, age at first live birth, duration of breast-feeding, age at menarche, ever OC use, education. Index dates 0.5 to 6.0 = exposure information was not considered 0.5, 2.0, 4.0, and 6.0 years prior to cancer diagnosis.

### Time-related exposure

Table 3 demonstrates the effect of duration of HRT use, of the time since first HRT use and time elapsed since last HRT use on risk of breast cancers; this table depicts only results at index date 0.5 year.

**Table 3:** Risk of breast cancer associated with HRT: Ever- use vs. never use of HRT.

	**e-case**^1^	**e-ctrl.**^1^	**Crude OR (95% CI)**	**Adj. OR (95% CI)**
**Ever HRT**	1492	3142	1.1 (0.99–1.2)	1.2 (1.0–1.3)
**Duration of use**^2^				
<2 yrs	387	770	1.3 (1.1–1.5)	1.3 (1.1–1.6)
2–4 yrs	223	670	0.8 (0.7–0.9)	0.9 (0.7–1.1)
5–7 yrs	278	609	1.0 (0.9–1.2)	1.1 (0.9–1.3)
8+ yrs	604	1093	1.2 (1.0–1.4)	1.2 (1.1–1.4)
**Time since first use**^2^			*P trend = .08*	*P trend = .06*
1–4 yrs	514	1187	1.1 (0.97–1.3)	1.2 (1.1–1.4)
5–9 yrs	556	1098	1.1 (0.98–1.3)	1.2 (1.0–1.4)
10–14 yrs	294	599	1.0 (0.9–1.2)	0.97 (0.8–1.2)
15+ yrs	128	258	1.1 (0.9–1.4)	0.96 (0.7–1.3)
**Time since last use**^2^			*P trend = .19*	*P trend = .51*
-1 yr/current use	1381	2908	1.1 (0.99–1.2)	1.2 (1.0–1.3)
1–2 yrs	71	162	0.96 (0.7–1.3)	0.9 (0.6–1.3)
3–4 yrs	31	63	1.0 (0.6–1.6)	1.1 (0.6–1.9)
5+ yrs	9	9	1.7 (0.6–4.3)	2.3 (0.7–7.2)
			*P trend = .14*	*P trend = .054*

Conditional logistic regression analysis [Odds ratios (OR) and 95% confidence intervals]; adjustment for BMI, family history of breast cancer, childbearing history, age at first live birth, duration of breast-feeding, age at menarche, ever OC use, education. Index dates 0.5 = exposure information was not considered 0.5 year prior to cancer diagnosis.

^1 ^e-case, e-ctrl = number of observations for exposed case or exposed control respectively.

^2 ^time variables were rounded

Duration of HRT use was apparently very little associated with breast cancer risk in our study. Two slightly increased risk estimates were observed (OR about 1.2, only marginally significant): duration up to 2 years and 8+ years. No significant trend of increasing breast cancer risk with increasing duration of HRT use was found.

Up to 10 years since first use the risk marginally increased in the two lower categories (up to 10 years until diagnosis). The risk estimates varied between 1.0 and 1.2 – and without trend of increasing risk with increasing time of diagnosis since first use.

Similarly, no trend of increasing cancer risk with increasing time since last HRT use was seen. The risk estimates were not significant and close to 1.0, with the exception of a non-significant cancer risk estimate for 5 years and more since last HRT use (small numbers).

### Effect modification

Established risk markers for breast cancer such as age at diagnosis (or at menarche, or first live birth), number of live births, breast feeding, OC use, BMI, family history of BC, or education were analyzed for modifying the association of HRT use and BC. All variables in Table 4 significantly modified the association between HRT use and BC in this study, although the risk differences between each of the two strata were apparently small. Only the BC risk of women with first-degree family history of BC and use of HRT was remarkably higher than in women without family history. Much less obvious effect modifications were seen comparing the two strata of age and ever use of OCs, i.e. higher BC risk associated with HRT use in women with higher age, and for women with no past history of OC use. In addition, there is evidence that the effect of HRT on BC risk is more pronounced in women with lower education level, higher BMI, earlier menarche, fewer births, but surprisingly also with prevalence of breast-feeding.

**Table 4:** Risk of breast cancer in HRT users stratified by established risk markers.

	Breast cancer			
	e-case^1^	e-ctrl.^1^	Crude OR (95% CI)	Adj. OR (95% CI)

Age at diagnosis				
<50 years	99	444	0.96 (0.7–1.2)	0.8 (0.6–1.1)
50+ years	1393	2698	1.1 (0.99–1.2)	1.3 (1.1–1.4)
Education			*P eff = 0.0*	*P eff = 0.0*
University level	455	1089	1.2 (0.96–1.6)	1.1 (0.7–1.5)
Lower education	1037	2053	1.2 (1.0–1.3)	1.3 (1.1–1.5)
Age at menarche			*P eff = 0.0*	*P eff = 0.0*
<13 years	764	1600	1.1 (0.9–1.3)	1.1 (0.9–1.3)
13+ years	728	1542	1.1 (0.9–1.3)	1.0 (0.8–1.3)
Child bearing history			*P eff = 0.0*	*P eff = 0.0*
0–1 child	678	1167	1.1 (0.9–1.3)	1.1 (0.8–1.4)
2+ children	814	1975	1.0 (0.9–1.2)	1.0 (0.9–1.2)
Age at first live birth			*P eff = 0.0*	*P eff = 0.0*
<= 22 years	593	1255	1.1 (0.9–1.3)	1.2 (0.9–1.5)
>22 years	700	1554	1.2 (0.97–1.4)	1.2 (0.95–1.5)
Breast-feeding			*P eff = 0.0*	*P eff = 0.0*
never	838	1552	1.1 (0.9–1.2)	1.1 (0.91–1.4)
1 month and more	654	1590	1.1 (0.96–1.4)	1.3 (1.0–1.6)
OC use			*P eff = 0.0*	*P eff = 0.0*
never	461	695	1.3 (0.99–1.6)	1.4 (1.0–1.8)
ever	1031	2447	1.2 (1.0–1.3)	1.1 (0.9–1.3)
Family history of breast cancer			*P eff = 0.0*	*P eff = 0.0*
No	1261	2871	1.1 (0.97–1.2)	1.1 (1.0–1.3)
Yes	231	271	5.3 (1.8–15.5)	6.4 (1.7–24.5)
Body mass index			*P eff = 0.0*	*P eff = 0.0*
<25	617	1546	1.2 (0.99–1.5)	0.99 (0.8–1.3)
25+	875	1596	1.0 (0.9–1.2)	1.2 (1.0–1.5)
			*P eff = 0.0*	*P eff = 0.0*

Conditional logistic regression: Odds ratios and 95% confidence intervals. Crude and adjusted for age, BMI, family history of breast cancer, childbearing history, age at first live birth, duration of breast-feeding, age at menarche, age at first OC use, education. Analyses for index date 0.5 year prior to cancer diagnosis.

^1 ^e-case, e-ctrl = number of observations for exposed case or exposed control respectively.

P eff = Significance for effect modification

To our surprise, there seemed to be a lower impact of HRT use on BC risk with increasing duration of past use of OCs, which needs further attention in future studies. The adjusted BC risk estimates were according to duration of OC use: never use [1.38 (1.04–1.84)], up to 2 years OC use [0.96 (0.39–2.34)], 2–4 years [not sufficient numbers for conditional analysis], 5–10 years [0.89 (0.32–2.41)], and for 10 and more years [0.72 (0.50–1.05)].

### Cancer risk of different HRT formulations

The aim of the analysis of different HRT formulation groups was to provide a crude, visual comparison of different groups of different HRT formulation across groups. The name of each formulation category in table 5 means that at least the labeled substance/combination was used sometime in life; all other formulation categories, however, could also have been used at different points in time. The categories are therefore not mutually exclusive, except the three categories of CEE/MPA combination.

**Table 5:** Risk of breast cancers associated with different categories of HRT formulation or administration.

	**Ever use**		**Duration of use**					
			**1–4 years**		**5–9 years**		**10+ years**	

**Formulation categories**	**e-cas/e-ctrl**	**Adj. OR (95% CI)**	**e-cas/e-ctrl**	**Adj. OR (95% CI)**	**e-cas/e-ctrl**	**Adj. OR (95% CI)**	**e-cas/e-ctrl**	**Adj. OR (95% CI)**

**A. Route of administration**								
oral	1053/2321	1.1 (0.99 – 1.3)	249/769	0.9 (0.7 – 1.1)	405/750	1.3 (0.7 – 1.1)	295/474	1.5 (1.2 – 1.9)
transdermal	211/650	0.8 (0.6 – 1.1)	60/212	0.7 (0.5 – 1.0)	81/189	1.1 (0.7 – 1.8)	52/161	0.9 (0.5 – 1.4)
other	94/307	0.7 (0.5 – 0.98)	23/98	0.5 (0.3 – 1.1)	24/78	0.6 (0.3 – 1.2)	28/81	0.7 (0.4 – 1.3)
**Only – HRTs**								
Estrogen (exclusive)	368/1168	0.8 (0.7 – 1.0)	82/341	0.7 (0.5 – 0.97)	140/365	0.99 (0.7 – 1.4)	107/311	0.8 (0.5 – 1.1)
Progestagen (exclusive)	108/321	1.0 (0.7 – 1.4)	30/104	0.8 (0.4 – 1.5)	29/74	1.8 (0.9 – 3.7)	35/75	0.96 (0.5 – 1.8)
**Combination – HRTs**								
Sequential formulations	546/1370	0.9 (0.8–1.1)	129/453	0.8 (0.6–1.1)	224/489	1.0 (0.8–1.3)	150/266	1.3 (0.96 – 1.9)
Continuous-combined	484/802	1.5 (1.2–1.8)	103/257	0.96 (0.7 – 1.4)	197/263	1.9 (1.4–2.6)	141/183	1.98 (1.4–2.9)
All E+P	916/1931	1.2 (1.0–1.3)	223/645	0.9 (0.7–1.0)	360/662	1.3 (1.0–1.6)	248/373	1.6 (1.2–2.0)
**CEE/MPA combinations**								
CEE + MPA	87/192	1.1 (0.7–1.7)	26/72	0.8 (0.4–1.6)	37/56	1.3 (0.7–2.7)	14/40	1.1 (0.4–3.1)
CEE or MPA	359/890	0.9 (0.7–1.2)	54/263	0.6 (0.4–0.99)	133/305	0.9 (0.7–1.3)	135/222	1.2 (0.9–1.8)
No CEE, no MPA	806/1723	1.2 (1.0–1.4)	224/611	0.94 (0.8–1.2)	293/517	1.4 (1.1–1.7)	194/306	1.6 (1.2–2.2)
**B. Formulation content**								
**Estrogen type**								
Estriol (E3)	12/40	0.4 (0.1 – 1.5)	1/11	n.d.	1/9	n.d.	6/8	0.4 (0.1 – 2.6)
Estradiol (E2)	889/2015	1.0 (0.9–1.2)	218/694	0.8 (0.7–1.0)	346/622	1.3 (1.1–1.4)	241/417	1.4 (1.1–1.7)
Ethinylestradiol (EE)*	11/41	0.8 (0.3–2.4)	4/15	n.d	4/6	n.d.	3/16	n.d.
Conjugated equine estrogens (CEE)	395/934	1.0 (0.8–1.2)	63/273	0.7 (0.5–1.0)	154/321	0.9 (0.7–1.3)	137/243	1.4 (0.98–2.0)
**Progestin type**								
Norethindrone acetate (NETA)	529/1037	1.2 (0.99–1.4)	114/346	0.8 (0.6–1.1)	209/338	1.4 (1.1–1.9)	157/218	1.9 (1.3–2.6)
Norgestrel (NG)	21/67	0.9 (0.4–2.2)	4/13	n.d.	7/28	0.6 (0.1–2.9)	6/19	0.7 (0.1–4.2)
LNG	290/562	1.3 (0.98–1.6)	69/177	1.2 (0.8–1.9)	131/227	1.4 (0.99–2.0)	69/109	1.3 (0.8–2.3)
Medroxyprogesterone acetate (MPA)	138/340	0.96 (0.7–1.3)	43/134	0.8 (0.4–1.3)	53/96	1.3 (0.7–2.3)	26/59	0.8 (0.4–1.7)
CMA	33/96	0.8 (0.5–1.5)	13/32	1.1 (0.4–3.1)	3/18	n.d.	12/21	0.7 (0.2–2.0)
CPA	33/116	0.6 (0.4–1.1)	11/54	0.5 (0.2–1.3)	11/22	1.2 (0.4–3.4)	7/18	0.6 (0.1–2.7)
Medrogestone	141/434	0.7 (0.5–1.0)	16/117	0.5 (0.2–0.9)	55/172	0.5 (0.3–0.9)	60/107	1.3 (0.8–2.2)
Dydrogesterone	9/51	0.5 (0.2–1.2)	2/20	n.d.	3/9	n.d.	2/12	n.d.
Progesterone	7/33	1.4 (0.4–4.4)	4/12	n.d.	1/6	n.d.	1/5	n.d.
Dienogest	10/24	0.9 (0.2–3.4)	1/12	n.d.	4/5	n.d.	3/3	n.d.
Tibolone	17/61	0.8 (0.3–2.1)	7/21	1.8 (0.4–8.3)	5/16	0.98 (0.1–7.9)	3/19	n.d.

The categories are not mutually exclusive – (see text). Conditional logistic regression: Odds ratios (OR) and 95% confidence intervals (95% CI); adjusted for age and residency. No OR was calculated if the frequency of exposed cases or controls was less than 5 women. This applied for use of HRTs containing desogestrel, lynestrenol, hydroxyprogesterone, and raloxifene. Only the index date 0.5 years is depicted in this table.

n.d. = no data.

* = in oral contraceptives used as HRT

The number of exposed cases and controls were very different across the subgroups of ever use of estrogens and progestins during lifetime. If numbers of exposed cases or controls were less than 5 (in any cell of the two-by-two table for risk estimation), the ORs were not calculated (labeled as n.d. = no data). Moreover, the loss of data during adjustment may leads in some instances also to the label "n.d.". Table 5 contains only risk estimates (and 95% confidence interval) for index date 0.5 year. In addition, the table is stratified by duration of use – to the extent numbers permitted ("rule of 5"- see above).

We distinguished between different routes of administration (oral, transdermal, and other), between therapy with exclusive use of estrogens or progestins throughout the entire lifetime (i.e. no other HRT was ever used), we considered two different estrogen/progestin combinations (sequential and continuous-combined), focused on the use of CEE and MPA specifically, and examined different estrogens and progestins.

The oral route of administration dominated in the participants of our study. HRT use of the oral route showed a mainly non-significant association with BC risk, which shows an increasing trend of risk with longer duration of use, i.e. becoming significant after 10 and more years of use. Transdermal use, however, did not show an increased risk of BC as did other forms of administration.

The BC risk associated with different formulation categories is small, and often not significantly different from never-use (as referent category = 1.0). Risk estimates for some formulation categories seem to be lower than unity (e.g., for estrogen only), others showed slightly, but significantly increased risk (e.g. continuous combined regimens) – if numbers are taken at face value.

Mono-therapies with estrogens or progestins seem to be different. Whereas exclusive estrogen use seems not to be associated with an increased risk of BC (even a non-significant tendency toward lower risk) than never use and depict no trend with increasing duration, "progestin-only" is also not significantly associated with BC risk but seems to show a trend of increasing risk with increasing duration of use.

Taken at face value, continuous combined regimens were associated with higher risk estimates (mostly significant) than sequential regimens (mainly non-significant). All E+P formulations together were associated with marginally higher BC risk estimates than estrogen- or progestagen-only formulations.

As far as CEE and MPA are concerned – no noteworthy increases in risk were evident if used combined or separately. Taking risk estimates at face value, there is no difference in risk when compared to all other formulations not containing CEE or MPA (confidence intervals largely overlapping).

Of the progestins, only NETA showed a slightly increased BC risk estimates. NETA showed – unlike all other progestins – an apparent upward trend with increasing duration of use. Small numbers and large confidence intervals should be considered.

## Discussion

Cancer of the breast is the most commonly occurring cancer in women, and the third most common cancer overall. Incidence and mortality for this and other gynecological cancers are still increasing in many parts of the world, especially in developed societies.

An expert panel from the American Institute of Cancer Research published an extensive review on "Food, Nutrition and the Prevention of Cancer", with consideration of all potential risk factors, beside others, also external use of hormones [21]. The panel included factors that probably act early in life, as well as others that might be lifestyle-related. Factors that increase the risk of breast cancer in particular include rapid early growth and greater adult height, but also a diet low in vegetables and fruit, alcohol consumption, weight gain in adult life, and high body mass after menopause [21]. The clearest non-dietary risk factors were those associated with hormonal and reproductive factors, such as nulliparity, late age at first pregnancy, late menopause, but also some inherited abnormalities that find expression e.g. in a positive family history. The mechanism by which hormonal factors may affect carcinogenesis at different gynecological sites was considered as unclear. It was thought that they may possibly play a promotional role [21]. The similarity of risk factors (including effects of exogenous hormones) across more or less all non-communicable diseases is striking and gives rise to speculation about non-specific, non-causal associations with gynecological tumors [22], for example.

Analysis of the effect of external hormones on gynecological cancer must take into account the lag time of cancer development. Lag time is likely to be long and may vary depending on complex unknown causal mechanisms, and is therefore an issue involving complex, time-dependent risk factors. It is difficult for observational studies to account sufficiently for time-dependent variables. Age, time, and duration of use are only indicator variables. Moreover, observational studies in general are prone to bias and residual confounding, even if their performance is "state-of-art". This is a methodological problem in particular, because observed risk estimates were usually small (less than or around 2.0 or 0.8 at the other side of the risk continuum), especially those associated with use of HRT/hormones. Given the great potential for residual confounding and bias, even a statistically significant small association might be inconclusive, because such associations could well be situated below reliable resolution levels of the "epidemiological microscope", i.e. it is not possible to discriminate between causation and bias/confounding [22,23].

Since most of the epidemiological studies before 2002 were performed in North America, and therefore primarily analyzed conjugated equine estrogens with or without MPA, we were interested in results for other HRT formulations more common in Europe; i.e. HRTs containing CEE were used by only 10% of our control and BC cases. Our register-based, case-control study showed marginally increased adjusted BC risk estimates of about 1.2 without variation with increasing lag time.

A review of the literature shows that an increased risk of breast cancer is often but not always reported in studies [12,13]. In a large-scale, pooled breast cancer review by the Oxford Group [13] containing 51 studies performed up to 1997, overall risk estimates for breast cancer and HRT ever-use in the individual studies ranged from 0.6 to 1.4 (often non-significant). In terms of exposure, HRT in this meta-analysis refers predominantly to conjugated equine estrogens (unopposed) in relatively high dosages. The overall summary risk was 1.14, and 1.34 after duration of at least 5 years. But there was also a group of 10 cohort studies with summary risk of 0.62 compared to never-use. Some reviews also indicate that the overall risk of HRT ever-use often shows no association [24,25]. More recent studies showed BC risk estimates of E+P users up to about 2fold increase.

Since HRT ever-use is a fairly crude summary indicator of exposure, it is advisable to look at time-related specifics such as duration of use and time since first and last exposures. We observed no significant trend toward risk with increasing duration of HRT use. Short-term use (under 2 years) and use of 8 and more years, however, was associated with a marginally significant increase in risk (see Table 3), whereas duration of use between 2 and 7 years showed no significant relation. Altogether, duration of use seems no convincing risk factor for BC. This has also been observed in other studies [26-28]. The possibility cannot be excluded that short-term risk estimates are biased, e.g. toward preferential detection of pre-existing breast cancer in HRT users shortly after the start of treatment, or differential misclassification of HRT exposure (never-use as opposed to very short duration). Obviously, the effect of duration of use was not homogeneous across different HRT formulations, particularly with respect to progestins.

The lack of a trend for duration of use – in aggregate – in our study is not congruent with the aggregate findings of the large-scale meta-analysis by the Oxford Group [13], in which the pooled risk estimate increased significantly with increasing duration of use of HRT (largely differing from the currently used formulations). BC risk estimates ranged from 1.5 to 2.3 after many years of HRT use in another review [24], but in one study the risk after 5+ years was 0.9 [29].

Time since first or last use of HRT seems not to play a role for breast cancer risk in our study; there is no significant trend with increasing time interval. However, the collaborative re-analysis by the Oxford Group did find such a trend [13]. It showed a significantly increasing trend of breast cancer risk with increasing time since first HRT use, and a decline in risk with increasing recency of use, although the change in point estimates over time was not impressive. However, due to the correlation among time variables, other significant time trends disappeared when duration and recency of use were taken into account [27].

The risk of BC associated with HRT was fairly consistent across strata of other established risk factors, i.e. all risk that we analyzed did show a significant effect modification. The differences in BC risk associated with HRT, however, were small or even not visible despite statistical significance in some variables such as age at menarche, child-bearing history, and age at first birth. The clinical relevance of this effect modification can therefore be questioned and the role of chance findings in so many sub-analyses cannot be ignored.

Some risk factors showed substantial effect modification such as age, education, breast-feeding, and first-degree family history of BC.

The effect of HRT use seems to be much stronger in women with a first-degree family history of BC in our study. Earlier results from the collaborative analysis by the Oxford Group [13] presented a different conclusion. The prevalence of first-degree family history is assumed to be 10–15% in BC cases and about 5% in controls [12]; our study fits into this picture. Internationally, however, the effects of family history of BC and use of external steroid hormones show numerous inconsistencies and paradoxes [12,13,30-33]. Many investigations have found that a positive family history amplifies the association between hormone use and BC risk, but others have observed no impact. Family history of BC, however, may not only reflect shared genes, but also shared environmental/lifestyle exposures. It is not trivial to disentangle both effects – even in family studies. There is definitely not enough evidence, however, to conclude that family history or genetic markers are the main causes of BC [12,30]. Thus, family history of BC and hormone treatment are among the many established factors and yet unknown agents that contribute to the risk of developing breast cancer.

HRT effect modification by former OC use seems to be of particular interest. We found an interesting significant impact from previous OC use, but there is little material published internationally on this topic. It seems that no use of OCs in the past increases the BC risk associated with HRT use, whereas former OC use showed no increased BC risk. This needs to be examined and either confirmed or refuted in further studies.

The association of different HRT formulations with BC risk suggested interesting results although the numbers were too small for deriving conclusions – at least for some of the progestin categories in our study.

We observed that the BC risk estimates were somewhat higher when HRT was used via the oral route than the transdermal route and became even significant after 10 years of use – which was not found for transdermal application. Another recently published study (E3N-EPIC cohort) showed also a slightly increasing risk with longer duration of HRT use via oral route, but not of transdermal administration [34]. However, the formulations are different in both ways of administration. Therefore it is important to analyze the BC risk by formulation groups. Other studies showed differences only when the formation was considered.

Our study confirmed that no increased BC risk or even decreased risk was associated with the use of estrogens alone. This fits in with the results of other studies [26,27], which have found no significant association or slight increase of risk [35].

The BC risk for all combined HRT formulations was slightly higher and increased with longer duration. The risk was lower for sequential than for continuous-combined formulations. Several recent studies have found similar results but often with higher risk estimates [7,24,26,36-38].

The BC risk associated with testosterone-derived progestins (e.g., NETA, LNG) seems – if taking the risk estimates at face value – to be slightly higher than with progesterone-derived progestins (e.g., MPA, CMA, CPA, medrogestone, dydrogesterone) on average but with a high variation at a low level of relative risk. However, to postulate a causal difference based on our study would be highly speculative – considering among others the fragile dataset of HRT formulation subgroups. It is far from clear, however, whether the apparently small difference in risk between progesterone- and testosterone-derived progestins is clinically meaningful. More general, it is debatable whether the apparent differences in BC risk across various progestins are real or simply chance findings due to multiple sub-analyses.

The Million Women Study [4] and the E3N-EPIC cohort [34] showed for HRT combinations of estrogens with progesterone- and testosterone-derived progestins increased BC risk estimates what seems not be supported by the low risk estimates associated with progesterone-derived progestins in our study.

In contrast, exclusive use of estrogens during lifetime seems not to be associated with an increased BC risk.

Also, no evidence was found to confirm our initial hypothesis that conjugated equine estrogens co-formulated with MPA might be associated with a different risk as opposed to "natural estrogens" with or without MPA.

More general, there is still uncertainty whether HRT use can really cause BC cancer induction de novo. A recent pathobiological review [39] made the convincing point that the development of cancers from the first malignant cell to a clinical diagnosis takes many years and therefore the biological development process is in apparent contradiction with results from epidemiological studies showing increased risk already after 1–6 years. For this and other reasons discussed, results of epidemiological studies should be interpreted with great caution.

### Limitations and strengths of the study

The cancer-register affiliated case-control study was based on voluntary participation. There are three main potential problem areas: self-selection, complex exposure biases, and recall bias. Although unlikely to differ across HRT formulation type, the self-selection of cases and controls to participate might be prompted by specific factors that affect the representativeness for the population. This cannot be excluded even for cases. Exposed subjects may have a higher likelihood of being selected into the study. The direction of this bias is unknown, because women might be motivated to participate either because they are particularly health-conscious, or because their health is poor, or because the used HRT, or for any number of other reasons.

We assume that the results of our study have not substantially affected by self-selection. Moreover women without tumor may more readily answer "never used HRT" instead of "short use only" in case they used HRT only a very short period of time as opposed to tumor cases. This exposure misclassification would lead to overestimated tumor risk estimates for short-term use – what we indeed observed in our study. The effect overlaps with recall bias: Women with tumors might better recall exposure to HRTs because they were asked this question frequently in the course of diagnosis and treatment. Women without tumors might never have been questioned about their HRT use or may not even know what it means. This would tend to increase the tumor risk associated with HRT use. Since the BC risk estimates were relatively low, we think that these biases were not dominant in our study.

Another potential source of bias is diagnostic suspicion bias. It cannot be assumed that tumor cases are equally identified (diagnosed) among HRT users and non-users. For instance, HRT users may be routinely advised by prescribing physicians to participate in screenings (e.g., repeated mammographies). Unfortunately, we could not control for the number of previous mammographies. This would lead to overestimated risk estimates. In addition, the possibility cannot be excluded e.g. that the women and/or their physicians were aware of "warning signs" (e.g. lumps, suspicious areas in imaging tests) that may have led to the decision not to use HRT in the year prior to final BC diagnosis. This exposure indication bias may have worked differentially, i.e. more often in later confirmed cancer cases than in controls. Stratification by index dates (increasing lag times) would help to identify the effect of such a bias that was not observed in our study. In most of our analyses we chose to disregard exposure at least 6 month prior to diagnosis/interview. Other authors decided to lag exposure by one year for the same reasoning [34].

An important paper was recently published closely related with this methodological discussion [11]. The WHI study provided evidence [11] that controlling for the time of initiating E+P use and confounding, the risk estimates became more similar between the WHI clinical trial and the WHI observational study. We used several approaches to consider time of exposure and confounding in our study based on lifetime history.

With regard to lifetime history of exposure, we were unable to compare mutually exclusive groups of different HRT formulations. Even if the hypothesis were acceptable that substantial differences in BC risk associated with different estrogens, progestins, and combinations would still be detectable despite dilution by overlapping exposure to different formulations during the lifetime, we cannot be certain. There is much room for speculation.

Despite some advantages of our study over many others in considering time-dependent exposure during the lifetime, or in introducing different lag times to perform stratified analyses, we don't know the impact of the above-mentioned biases on the observed risk estimates. Our interpretation of the low BC risk estimates in our observational study and of the high potential for residual confounding and bias is that we cannot distinguish between causality and bias/confounding. In other words, the slightly increased BC risk estimates associated with HRT use might well be explained by bias and confounding despite all efforts to account for them. On the other hand, we cannot exclude a slightly increased risk of breast cancer associated with use of HRT either. In addition, we also cannot exclude a slightly higher BC risk for users of continuous-combined than of sequential formulation although the differences were small, i.e. possibly too small to be clinically relevant and too much room for bias to be causal.

## Conclusion

Taking the results at face value, ever-use of HRT is associated with a slightly increased risk of breast cancer. No significant trend of increasing BC risk with increasing duration of HRT use was observed in aggregate. There was, however, some evidence that the risk of BC might increase with longer use of some HRT formulation groups. Neither time since first use nor time since last use of HRT showed any association with BC risk. Many established BC risk factors significantly modified the effect of HRT on BC risk. Apparently, BC risk did not vary markedly among different HRT formulations (estrogens, progestins). This should be interpreted with great caution because of mutually not exclusive HRT user profiles (subgroups) and small numbers. However, there might be differences that were not detectable in our study due to the "limited solution of the epidemiological microscope".

We do not feel comfortable distinguishing between causal associations of BC risk and HRT use in our study and the effects of residual confounding and bias.

## Competing interests

A pharmaceutical company that produces HRT products financially co-sponsored this study.

## Authors' contributions

JCD: contributed to design the study, wrote the first draft of the manuscript, and was involved in the data analysis; LAJH: responsible for the design the study and prepared the data analysis plan, responsible for writing the manuscript; SM: responsible for the collaboration with the tumor centers and quality checks and management of the initial database, contributed to the manuscript; DMT: responsible for final data management and data analysis; contributed to the manuscript; AA: responsible for development of all questionnaires (together with SM), contributed to organizing field work and quality checks, contributed to the manuscript.

## Pre-publication history

The pre-publication history for this paper can be accessed here:


